# Effect of acupuncture on episodic memory for amnesia-type mild cognitive impairment: study protocol of a multicenter, randomized, controlled trial

**DOI:** 10.1186/s12906-023-04059-9

**Published:** 2023-07-28

**Authors:** Yalan Dai, Rui Xia, Dan Wang, Shuqian Li, Xu Yuan, Xingjie Li, Jun Liu, Mengyang Wang, Yuxing Kuang, Shangjie Chen

**Affiliations:** 1 Department of Rehabilitation Medicine, The People’s Hospital of Baoan Shenzhen, Shenzhen, China; 2 College of Rehabilitation Medicine, Fujian University of Traditional Chinese Medicine, Fuzhou, China; 3 Department of Rehabilitation Medicine, The Second Affiliated Hospital of Shenzhen University, Shenzhen, China

**Keywords:** Acupuncture, Amnesic mild cognitive impairment, Episodic memory, Randomized controlled trial, Protocol

## Abstract

**Background:**

Amnesic mild cognitive impairment (aMCI) is the main subtype of mild cognitive impairment (MCI) and has the highest risk of conversion to Alzheimer’s disease (AD) among all MCI subtypes. Episodic memory impairment is the early cognitive impairment of aMCI, which has become an important target for AD prevention. Previous clinical evidence has shown that acupuncture can improve the cognitive ability of MCI patients. This experiment aimed to observe the efficacy and neural mechanism of *TiaoshenYizhi* acupuncture on the episodic memory of patients with aMCI.

**Methods:**

In this multicenter, parallel-group, double-blind, randomized controlled trial, 360 aMCI participants will be recruited from six subcenters and randomly assigned to the acupuncture group, sham acupuncture group, and control group. The acupuncture group will receive *TiaoshenYizhi* (TSYZ) acupuncture, the sham acupuncture group will use streitberger sham acupuncture, and the control group will only receive free health education. Participants in the two acupuncture groups will receive real acupuncture treatment or placebo acupuncture three times per week, 24 sessions over 8 consecutive weeks. The primary outcome will be global cognitive ability. Secondary outcomes will be a specific cognitive domain, including episodic memory and execution ability, electroencephalogram, and functional magnetic resonance imaging data. Outcomes will be measured at baseline and the fourth and eighth weeks after randomization. Repeated measurement analysis of variance and a mixed linear model will be used to observe the intervention effect.

**Discussion:**

The protocol will give a detailed procedure to the multicenter clinical trial to further evaluate the efficacy and neural mechanism of *TiaoshenYizhi* acupuncture on episodic memory in patients with aMCI. From this research, we expect to provide clinical evidence for early aMCI management.

**Trial registration:**

http://www.chictr.org.cn/edit.aspx?pid=142612&htm=4, identifier: ChiCTR2100054009.

**Supplementary Information:**

The online version contains supplementary material available at 10.1186/s12906-023-04059-9.

## Background

Mild cognitive impairment (MCI) is the precursor stage of Alzheimer’s disease (AD), mainly manifested as memory or cognitive impairment that has no significant effect on daily ability [1]. The latest epidemiological survey results have shown that 38.77 million people aged 60 years and above in China suffer from MCI [2]. Among all subtypes of MCI, amnesic MCI (aMCI) has become a research hotspot because of its high incidence rate, high prevalence, and high conversion rate to AD. A meta-analysis of 41 studies on Chinese community-dwelling populations over 55 years of age has reported that the pooled prevalence of MCI was 12.2% for MCI and 10.9% for aMCI [3]. In a recent random sampling survey of 3246 community-dwelling older people in China, the prevalence of aMCI has been 17.1%, with an annual incidence rate of 70.57% [4]. Although MCI is a transitional state between normal aging and AD, its prognosis may have different outcomes due to its heterogeneity [5]. Studies have shown that the AD conversion rate is higher in aMCI than in the non-amnestic type, and aMCI is the subtype of MCI predominantly progressing to AD [6–8]. Therefore, it is of great significance to explore the early treatment of aMCI.

As a commonly used and safe complementary and alternative therapy, acupuncture has been demonstrated to be potentially effective in improving cognitive function in patients with MCI and dementia [9–15]. However, many existing studies have not distinguished non-amnestic MCI from aMCI. The symptoms of aMCI primarily comprise a decline in memory function with other cognitive domains remaining relatively intact, while the non-amnestic MCI does not cause memory impairment as the first symptom and prominent feature [8, 16, 17]. There are some specific biomarkers for the early diagnosis of aMCI and AD, such as β-amyloid and tau, but these measurements in clinical screening are invasive and expensive [18]. Episodic memory-associated neuropsychological scale screening is mature and inexpensive. Episodic memory impairment is the earliest and core manifestation of AD and a landmark clinical symptom of aMCI [19–23]. More evidence is needed to determine in what way and to what extent acupuncture could influence the episodic memory of patients with aMCI.

Most of the research on acupuncture treatment of MCI has focused on general cognitive function. There are many indexes in Mini-mental State Examination (MMSE), Montreal Cognitive Assessment Scale (MoCA), and Behavioral Observation Scales (BOS), which are closely related to episodic memory [23, 24]. These studies have also shown that acupuncture might have a positive effect on episodic memory, but there is a lack of special evaluation of episodic memory; thus, the research has not been in-depth. Therefore, it is extremely significant to investigate acupuncture intervention for the early episodic memory disorder of patients with aMCI.

### Objectives

The main purpose of this multicenter study is to verify the efficacy and safety of acupuncture treatment for episodic memory impairment in patients with aMCI through a randomized controlled trial and then explore its neuroimaging mechanism through functional magnetic resonance imaging (fMRI) and electroencephalogram (EEG).

## Methods and analysis

### Study design

**Fig. 1:**
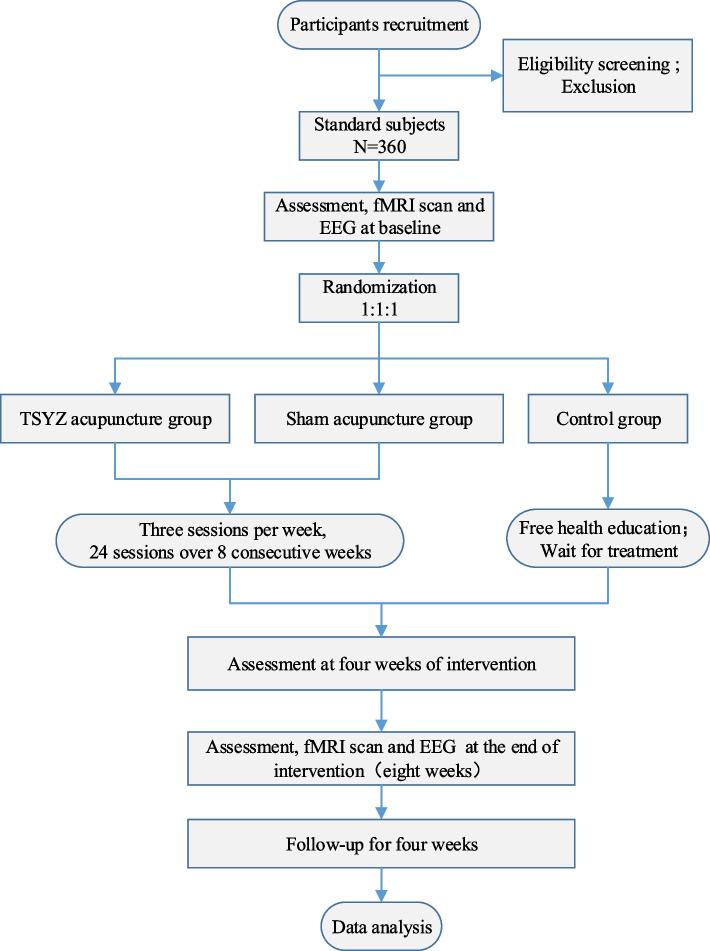
Flow chart of the study

The design of this study is a randomized controlled trial, strictly following the Standard Protocol Items: Recommendations for Interventional Trials (SPIRIT) statement (see Additional file 1) [25]. The allocation will be hidden, and the allocation ratio will be 1:1:1. A total of 360 eligible participants will be randomly assigned to the TiaoshenYizhi acupuncture group, the sham acupuncture group, and the control group. Participants will be evaluated at baseline (weeks -2 to 0) and weeks four, eight, and 12. EEG will be used to explore the diagnostic value of event-related potential (ERP) in aMCI, and fMRI will be used to measure the structure and function of brain regions related to episodic memory at baseline and eight weeks. All data collectors will not know the group assignment. The research procedure and results evaluation schedule of this trial are shown in Fig. 1 and Table 1.

**Table 1:** Location of acupoints

Acupoints	Locations	Insert depth
Sishencong (EX-HN1)	On the vertex, 1 cun from the front, back, left, and right to GV20, common 4 points	0.5 cun
Yintang (EX-HN3)	At the forehead, at the midpoint between the two medial ends of the eyebrow	0.5 cun
Neiguan (PC6) (bilateral)	On the palmar aspect of the forearm, 2 cun above the transverse crease of the wrist, on the line connecting PC3 and PC7, between the tendons of m. palmaris longus and m. flexor carpi radialis	1.0 cun
Fenglong (ST40) (bilateral)	On the anterior aspect of the lower leg, 8 cun superior to the external malleolus, lateral to ST38, two fifinger-breadth (middle fifinger) from the anterior crest of the tibia	1.5 cun
Sanyinjiao (SP6) (bilateral)	On the medial side of the shank, 3 cun above the medial malleolus, by the posterior of the tibia	1.5 cun
Taixi (KI3) (bilateral)	On the medial border of the foot.posterior to the medial malleolus, in the depression between the tip of the medial malleolus and Achilles tendon	0.5 cun
Taichong (LR3) (bilateral)	On the dorsum of the foot, in the depression proximal to the fifirst metatarsal space	0.5 cun

### Study population

The study population will be community-based aMCI patients in Shenzhen, Fuzhou, Nanning, Nanjing, Bengbu, and Jiujiang city. The diagnostic, inclusion, and exclusion criteria of the study sample are as follows.

### Eligibility

#### Diagnostic criteria

The diagnostic criteria refer to the 2003 International Working Group’s diagnostic criteria for MCI recommended by the 2018 Chinese guidelines for the diagnosis and treatment of dementia and cognitive impairment, and the aMCI subtype will be determined based on these criteria [17, 26].

#### Inclusion criteria

The eligible participants must meet the following criteria:

1. Participants meeting the diagnostic criteria of aMCI.
2. Participants aged 55–75 years.
3. The participants’ mother tongue will be Chinese, and they will have at least a primary school education.
4. Participants will be right-handed.
5. Participants who have subjectively ongoing memory decline or cognitive decline compared to their normal status within the last five years, as confirmed by their caregivers.
6. Participants whose ability of daily living (ADL) remained normal with ADL scale (14-item table) scores being ≤ 18 points.
7. Participants participate voluntarily and provide signed informed consent.

#### Exclusion criteria

The following exclusion criteria will be used.

1. People with metal residues and tattoos on the body.
2. People afraid of fMRI or those who cannot be scanned by fMRI for other reasons.
3. People with identified or suspected brain lesions.
4. People with a history of heavy drinking and drug abuse.
5. People with a history of mental illness or congenital mental retardation; people who are taking anti-anxiety or anti-depression drugs and have been receiving psychological treatment in the past three months.
6. People with serious heart, liver, and kidney diseases.
7. People with other diseases of the nervous system that can cause cognitive dysfunction, such as brain tumors, encephalitis, epilepsy, Parkinson’s disease, etc.

### Recruitment

Participants will be recruited from the community of the six centers through WeChat ads, flyers, posters, volunteer recommendations, and community publicity. Potential eligible individuals will first complete the screening of community doctors in the community health service center, and their qualifications will be determined according to the inclusion and exclusion criteria. Qualified personnel interested in participating in the study will have an informed discussion with trained research assistants. The research assistant will obtain written informed consent from individuals willing to participate before the baseline assessment.

### Randomization, allocation concealment, and blinding

After baseline evaluation, the eligible participants will be randomly divided into the TiaoshenYizhi acupuncture group, sham acupuncture group, and control group. The randomly assigned sequences will be generated using the online randomization tool MedSci (http://tools.medsci.cn/rand) and managed by independent statisticians who are not involved in the recruitment, evaluation, and intervention of participants. The independent research assistant will inform eligible participants of their allocation results by telephone. We could not establish the blinding of participants, acupuncturists, or intervention supervisors to the assigned treatment, but result evaluators and data statisticians will be blind to the grouping assignment.

### Interventions

#### TSYZ acupuncture group

**Fig. 2:**
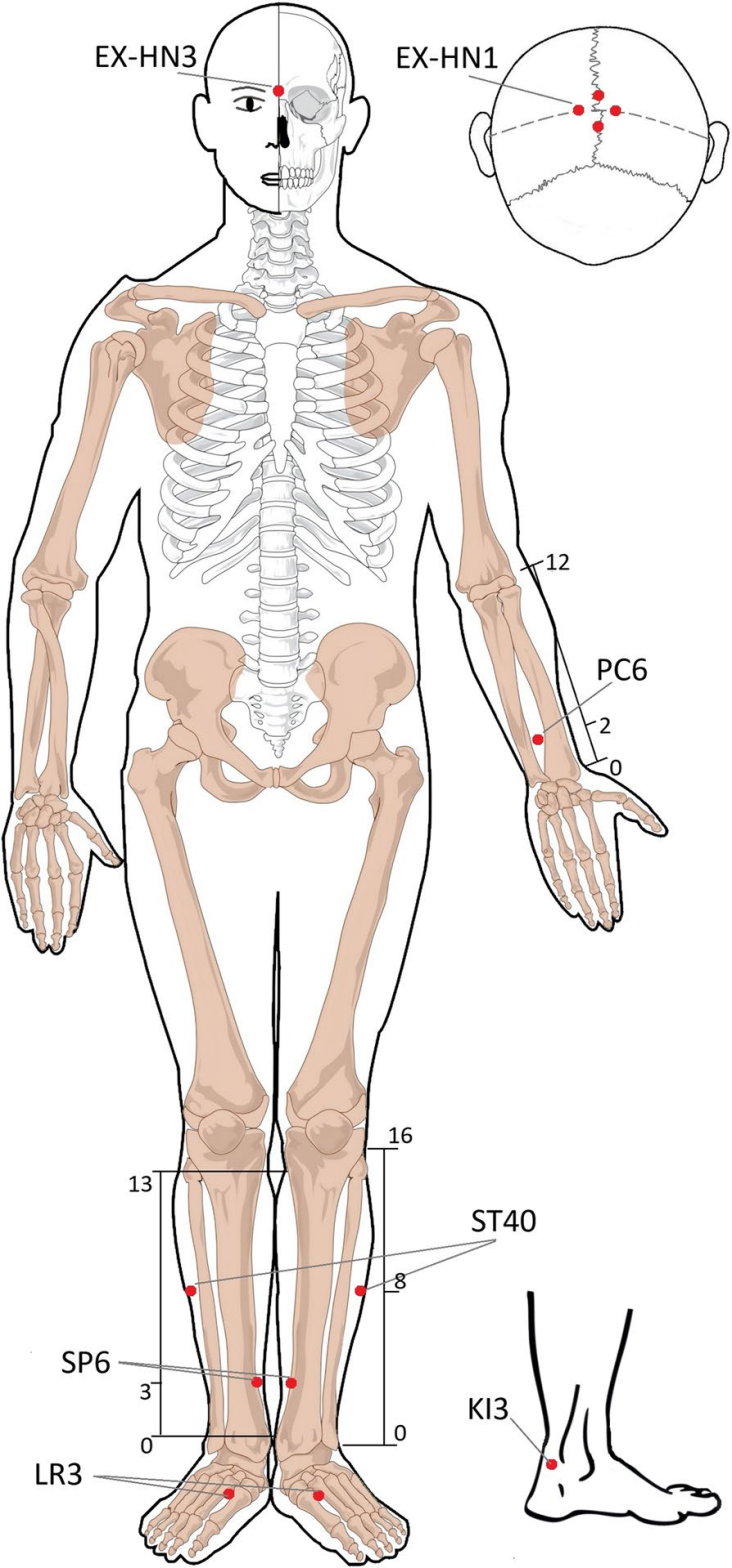
Location of acupoints

Patients allocated to the TiaoshenYizhi (TSYZ) acupuncture group will take treatment with needles inserted at the prespecified acupuncture points. The acupoints include Sishenchong (EX-HN1), Yintang (EX-HN3), Neiguan (PC6), Fenglong (ST40), bilateral Sanyinjiao (SP6), Taixi (KI3), and Taichong (LR3). All acupoints are localized according to the WHO Standard Acupuncture Locations and exhibited in Fig. 2 and Table 1. Manipulations of twirling, lifting, and thrusting will be performed on all needles for at least 30 s to reach De qi (a compositional sensation including soreness, numbness, distention, and heaviness), which is believed to be an essential component for acupuncture efficacy [27].

#### Sham acupuncture group

**Fig. 3:**
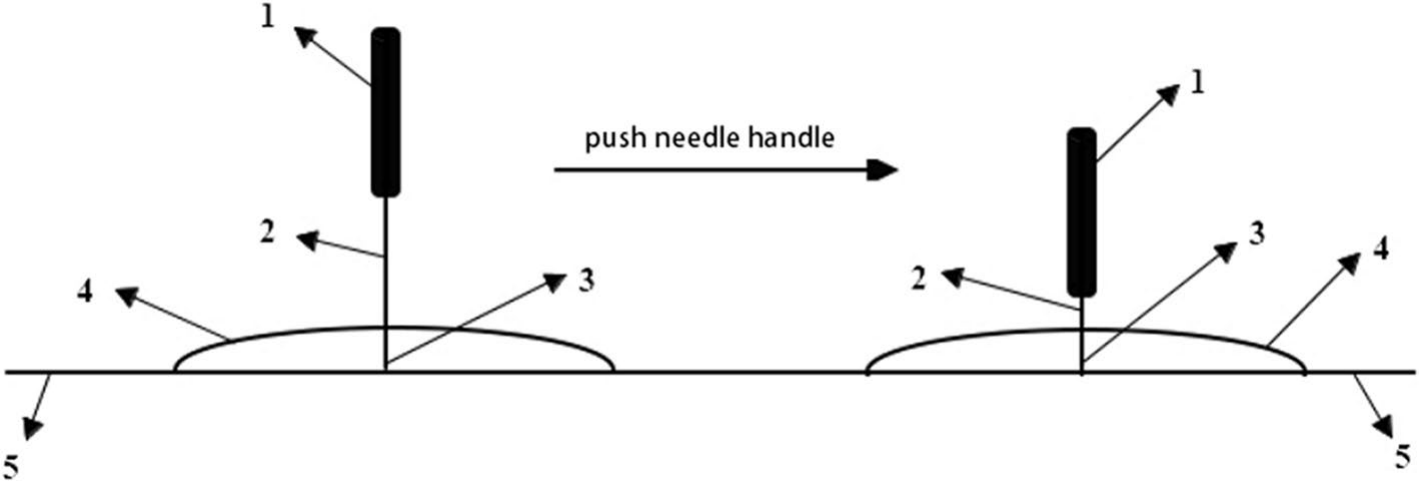
Diagram of the streitberger sham acupuncture. 1. Needle handle. 2. Needle. 3. The blunt tip of the placebo needle. 4. Plastic cover. 5. Skin

The streitberger sham acupuncture method will be used [28]. The acupuncturist will use a placebo needle in the sham acupuncture group. This placebo needle is not fixed inside the handle, and its tip is blunt. When the blunt needle touches the skin, a pinprick-like sensation will be felt by the person, while the needle is actually retracted into the handle without penetrating the skin and appears to be shortened (Fig. 3).

The same acupoints as those in the TiaoshenYizhi acupuncture group are selected for placebo needle stimulation to avoid a site-specific effect of acupuncture. The angulation, duration of acupuncture manipulation, and the shortening of the needle will also appear the same as in the TSYZ acupuncture group.

#### Control group

The participants in control group will receive health education with a frequency of one session every 4 weeks (40 min per session) like the acupuncture groups.

### Outcome assessment

The variables in this trial consist of demographics and clinical characteristics, primary outcomes, and secondary outcomes. The basic characteristics will be measured at baseline (1–2 weeks before randomized allocation). Primary and secondary outcomes will be measured at baseline, the middle of intervention (4 weeks), the end of intervention (8 weeks), and 4 weeks of follow-up. Outcomes of fMRI and EEG will be measured at baseline and the end of intervention (8 weeks). All outcomes will be evaluated by experienced doctors who will be blinded to the allocation results of participants. A summary of all measurements in this trial is shown in Table 2.

**Table 2:** Trial processes chart

Study period Item	Enrolment and Baseline	Intervention Period (8 weeks, one time per 4 weeks)				Follow-up phase
**Week**	-2 to 0	1 to 3	4	5 to 7	8	12
**Enrolment**						
Inclusion criteria	×					
Exclusion criteria	×					
Informed consent	×					
**Intervention**						
TSYZ acupuncture group		×	×	×	×	
Sham acupuncture group		×	×	×	×	
Blank control group						
**Outcome measurement**						
GDS	×					
BDI	×					
MoCA	×		×		×	×
AVLT	×		×		×	×
TMT	×		×		×	×
DSST	×		×		×	×
SF-36	×		×		×	×
fMRI scan	×				×	
EEG	×				×	
**Safety**						
Adverse events		×	×	×	×	×
Reasons for dropout and withdrawals		×	×	×	×	

*TSYZ* Tiaoshenyizhi, *GDS* Global Deterioration Scale, *BDI* Beck Depression Inventory, *MoCA* Montreal Cognitive Assessment, *AVLT-H* Auditory Verbal Learning Test-Huashan version, *TMT* Trail Making Test, *DSST* Digit Span Subtest, *SF-36* Medical Outcomes Study 36-item short form health survey, *EEG* Electroencephalogram, *fMRI* Functional magnetic resonance imaging

#### Basic characteristics

Participants’ demographic characteristics (e.g., sex, age, education, marital status, living arrangements, occupation, and socioeconomic status) and history of disease and medication use will be collected by the recruiters using the self-designed questionnaire. The basic MoCA score, Global Deterioration Scale (GDS), and Beck Depression Inventory (BDI) will be used to assess the corresponding characteristics. Baseline measurement will be completed before randomization.

#### Primary outcomes

Cognitive function will be measured using the MoCA scale, which is a cognitive screening instrument created and validated to detect MCI. MoCA is a brief (about 10 min) test evaluating visuospatial/executive functions, naming, verbal memory registration and learning, attention, abstraction, 5-min delayed verbal memory, and orientation with a total score of 0–30 (a higher score equals better function). The Chinese version of MoCA (Fuzhou version) will be applied to this trial with demonstrated good validity, reliability, and sensitivity in the population in Fuzhou city [29].

#### Secondary outcomes

Episodic memory will be evaluated using the Auditory Verbal Learning Test-Huashan version (AVLT-H). AVLT-H assesses the short-term delayed free recall and the long-term delayed free recall, having good reliability and validity in evaluating episodic memory of patients with aMCI [30]. The participant will be asked to repeat learning a list of 12 words three times, with a short delayed recall after an interval of 3–5 min, and a long delayed recall, cue recall and identification after an interval of 20 min. The most sensitive evaluation index is the score of long delay recall and identification. The score of the AVLT-H will be expressed as a memory quotient (MQ), and the higher the MQ means better memory.

The execution function will be assessed using the Chinese version of Trail Making Test-B (TMT-B) [31, 32]. In TMT-B test, the participant will be asked to draw a line alternating between circles and squares according to the numbers in ascending order. The maximum time of completion for each participant is 300 s. Attention ability and short-term memory (STM) will be measured using the Digit Span Subtest (DSST) from the Wechsler Adult Intelligence Scale-IV.

Quality of life will be measured by the Medical Outcomes Study 36-Item Short-Form Health Survey (SF-36) [33]. It consists of 36 items assessing eight health concepts: functional capacity (10 items), physical activities (4 items), social activities (2 items), bodily pain (2 items), mental health (5 items), usual activities (4 items), vitality (4 items), and general health status (5 items) [34]. Each health concept is evaluated separately by the normalized scores of 0–100, with 0 corresponding to the worst health status and 100 corresponding to the best health status. The Chinese version of SF-36 has demonstrated high reliability and validity in the Chinese population [35, 36].

#### fMRI and EEG outcomes

The function of related brain regions will be measured using fMRI. The fMRI scan will be conducted in the medical imaging department of Rehabilitation Hospital Affiliated with Fujian University of Traditional Chinese Medicine. Siemens Prisma 3.0T MR scanner and Siemens 64-channel head and neck coil will be used for fMRI data acquisition. Scanning parameters are as follows:

- High-resolution three-dimensional T1-weighted: TR = 2300 ms, TE = 2.27 ms, flip angle = 8°, slice thickness = 1.0 mm, FOV = 250 × 250 mm, matrix = 256 × 256, Voxel size = 0.98 × 0.98 × 1 mm^3^, and number of slices = 160.
- Resting fMRI: TR = 2000 ms, TE = 30 ms, flip angle = 90°, slice thickness = 3.6 mm, FOV = 230 × 230 mm, matrix = 64 × 64, voxel size = 3.6 × 3.6 × 3.6 mm^3^, number of slices = 37, axial slices = 35, and phases per location = 240.

EEG measurements will include different frequency bands (front α, α, β, δ, and θ) of spectral power and θ/α ratio. Receiver operating characteristic (ROC) analysis will be performed to evaluate the accuracy of EEG as a diagnostic indicator.

#### Safety measurements

Any possible accidental injuries caused by this study will be prevented as much as possible, the relevant medical expenses will be covered by the research project team, and the participants will receive certain financial compensation. Any unexpected adverse events during the intervention period will be monitored and reported to a research assistant. Causality in relation to the acupuncture intervention and the severity of adverse events will be analyzed. Serious adverse events will be reported to the ethics committee.

### Sample size

MoCA score of the cognitive function assessment scale will be used as an effect index to evaluate the recovery of cognitive function in patients with MCI. According to the previous study and clinical experience, it has been estimated that the MoCA score difference between the elderly with MCI and the baseline after eight weeks of acupuncture was 5.50 ± 2.48 points, and the MoCA score difference of the control group was 4.88 ± 2.45 points [37]. On this basis, the calculated effect quantity is 0.17, and by the two-sided test, α = 0.05, β = 0.20, calculated by gpower 3.1.9.2 software, and the sample size is 300 cases. Considering the loss of follow-up rate of 20%, the total sample size of this study is 360 cases, i.e., 120 cases in each group.

### Statistical analysis

Statistical analysis will be performed using SPSS software V.26.0 (IBM) package by an independent statistician, with a statistical significance of *P* value < 0.05 on both sides. Categorical variables will be described as frequencies or percentages using a chi-square test. For the normal distribution, continuous variables will be expressed using mean ± standard deviation (SD) and will be assessed by one-way analysis of variance (ANOVA) and compared between every group. For non-normal distribution, continuous variables will be expressed using the median and its interquartile range (IQR) and be analyzed by the rank sum test. The missing data will be filled in using the multiple imputation method. When baseline index imbalances occur among the three groups, a general linear model or logistic regression model will be applied for the calibration and analysis.

Adverse effects (AEs) will be analyzed using a Chi-square test or Fisher’s exact test. If the formal statistical analyses between groups cannot be performed due to the lack of power, AEs will be tabulated and summarized using descriptive statistics. For fMRI data, DPABI (a toolbox for Data Processing & Analysis for Brain Imaging, Version 2.3_170105, http://rfmri.org/dpabi) software package will be used to preprocess the image data obtained by fMRI scanning under the Matlab2013a (The Match Works, Inc). Independent component analysis (ICA) will be used to extract time signals for sequence dynamic causal modeling (DCM) analysis. Components will be extracted from the preprocessed images using GIFT v4.0 (http://icatb.sourceforge.net/).

## Discussion

Evidence has indicated that the pathological and physiological alterations of AD may appear years earlier than the clinical symptoms. In the diagnostic guidelines for AD issued in the US, aMCI fulfilled the clinical diagnostic criteria of the presymptomatic stage before dementia [38–40]. When compared to AD patients, patients with aMCI exhibit impaired autobiographical memory characterized by decreased episodic memory function and stronger semantic memory [41]. The impairment of episodic memory in aMCI is closely related to the functional connectivity of multiple brain networks [16, 42, 43]. Therefore, in this study, fMRI scans will be performed before and after the intervention to explore the neuroimaging mechanism of TiaoshenYizhi acupuncture and how to influence episodic memory in patients with aMCI.

The original acupuncture prescription of *TiaoshenYizhi* composed of the six acupoints Sishencong (EX-HN1), Yintang (EX-HN3), Neiguan (PC6), Fenglong (ST40), Taixi (KI3), and Taichong (LR3) has been based on actual clinical experience and our previous experiment [44]. Moreover, our previous studies have shown that the Taixi (KI3) acupoint probably can activate some brain regions and have relatively functional specificity of acupuncture [45, 46]. In the process of research and clinical practice, experienced experts of acupuncture suggest that Sanyinjiao (SP6) could be added. According to evidence, Sanyinjiao (SP6) acupoint is one of the most-used acupoints for the intervention of various cognitive impairments; hence, we adopted the advice of experts and determined the current acupoint selection of *TiaoshenYizhi* [47–49]*.*

The characteristics of acupuncture treatment make it difficult to implement the double-blind method; therefore, we will blind participants by setting up the sham acupuncture group as a placebo [28, 50]. Considering the placebo effect of sham acupuncture, the health education group will be set up as a negative control group, providing prevention knowledge of common geriatric diseases, including AD. We increased the sample size and expanded the scope of the crowd compared with our previous research. The intervention can be implemented in community medical centers, and the adverse events can be fully evaluated. The main challenge of this study is whether such a large number of patients with aMCI are willing to receive acupuncture treatment until the end of the study and cooperate with the collection of each outcome. We will give material rewards to the participants.

In conclusion, through this study, the effectiveness of TiaoshenYizhi acupuncture on the episodic memory of patients with aMCI will be further clarified. We expect that this study will provide a reference for early treatment of aMCI and AD. The results of the study will be reported according to the guidance of the Consolidated Standards of Reporting Trials statement (STRICTA) [51].

### Trial status

Participant recruitment is in progress. We have recruited 37 participants on Aug. 12, 2022.

## Supplementary Information

**Additional file 1.** SPIRIT 2013 Checklist: Recommended items to address in a clinical trial protocol and related documents*.

## Data Availability

Not applicable; no data have yet been generated.

## Abbreviations

aMCI: Amnesic mild cognitive impairment
MCI: Mild cognitive impairment
AD: Alzheimer's disease
EEG: Electroencephalogram
fMRI: Functional magnetic resonance imaging
MoCA: Montreal Cognitive Assessment Scale
MMSE: Mini-mental State Examination
SPIRIT: Standard Protocol Items-Recommendations for Interventional Trials
ERP: Event related potential
GDS: Global Deterioration Scale
BDI: Beck Depression Inventory
AVLT-H: Auditory Verbal Learning Test-Huashan version
MQ: Memory quotient
TMT: Trail Making Test
STM: Short-term memory
DSST: Digit Span Subtest
SF-36: Medical Outcomes Study 36-item short form health survey
ROC: Receiver operating characteristic
ICA: Independent component analysis
DCM: Dynamic causality model
AEs: Adverse effects
CONSORT: Consolidated Standards of Reporting Trials statement
CRF: Case report form
EDC: Electronic data acquisition system
